# Anthropomorphic technology in everyday life: focus on chatbots and impacts on mental health

**DOI:** 10.1007/s00406-025-02088-8

**Published:** 2025-10-08

**Authors:** Scott Monteith, Tasha Glenn, John R. Geddes, Peter C. Whybrow, Eric Achtyes, Michael Bauer

**Affiliations:** 1 Michigan State University College of Human Medicine, Traverse City Campus, 1400 Medical Campus Drive, Traverse City, MI 49684 USA; 2 ChronoRecord Association, Fullerton, CA USA; 3 Department of Psychiatry, University of Oxford, Warneford Hospital, Oxford, UK; 4 Department of Psychiatry and Biobehavioral Sciences, Semel Institute for Neuroscience and Human Behavior, University of California Los Angeles (UCLA), Los Angeles, CA USA; 5 Department of Psychiatry, Western Michigan University Homer Stryker M.D. School of Medicine, Kalamazoo, MI USA; 6 Department of Psychiatry and Psychotherapy, University Hospital Carl Gustav Carus Medical Faculty, Technische Universität Dresden, Dresden, Germany

**Keywords:** Chatbots, Anthropomorphism, Artificial intelligence, Mental health

## Abstract

Chatbots are widely used by business in everyday life to interact with consumers in e-commerce, social networking, education, and government settings. Anthropomorphism is a fundamental aspect of chatbot design and implementation, and increases the interaction of chatbots with humans. As part of the recent expansion of artificial intelligence (AI) technologies, chatbots incorporated AI to engage more personally with consumers. The anthropomorphic characteristics of chatbots, including both visual appearance and language, influence the interaction of chatbots with humans in diverse ways that may have both positive and negative consequences. The purpose of this review is to increase physician awareness of the use of chatbots which use AI to enhance anthropomorphism. Consumers routinely anthropomorphize products including chatbots. Physicians need to be aware of the wide-ranging and routine use of chatbots, and the importance of anthropomorphism in chatbot design and implementation. Physicians also need to recognize potential negative consequences of anthropomorphism of chatbots on mental health.

## Introduction

Consumers routinely anthropomorphize the products they interact with, attributing human characteristics to them including names, knowledge, intentions, beliefs, and capabilities [1, 2]. Commerce has long recognized this behavior, and routinely encourage anthropomorphism of brands and products by consumers when marketing goods and services, including in healthcare [3]. For example, many animated spokes-characters have been included in direct-to-consumer marketing of pharmaceuticals in the US [4, 5]. Anthropomorphism is viewed as a fundamental aspect of developing consumer brand loyalty [6]. For consumers, anthropomorphism of marketing messages increases both the feeling of connection, and comprehension of the products being sold [3]. The widespread use of chatbots that use artificial intelligence (AI) and directly interact with consumers have greatly increased the opportunities to market healthcare products by encouraging consumer anthropomorphism. The purpose of this paper is to review the potential consequences of the increasing anthropomorphic messaging that is received by consumers in the era of AI, with an emphasis on how chatbots in daily life outside of healthcare may affect mental health.

## Recent technology advancements

During the last decade, the use of AI technologies in everyday life has greatly expanded. Diverse techniques such as machine learning for predictive analysis, natural language processing, rule-based expert systems, generative AI including large language models that produce original content, and the creation of AI agents or chatbots have all been employed to engage with customers [7, 8]. The use of AI technology creates new opportunities and challenges different from those found with traditional technologies [7]. The global chatbot industry is expected to expand at an annual growth rate of 23.3% from 2024 to 2030 [9]. The varied businesses using chatbots include online e-commerce, social networking, education, banking, music streaming and the travel industry, with the chatbots often providing customer support [10, 11]. Chatbots have also been widely adopted by governments to address the information and service requests from citizens [12]. Consumers anthropomorphize chatbots in large part because companies design chatbots to encourage anthropomorphism.

## Background on chatbots

Chatbots are a type of computer program that communicate with a user in friendly verbal or text conversations, giving the impression of human intelligence [13–15]. The goal for the chatbot is to make the person feel as if they are conversing with another human [15]. One of the earliest chatbots was Eliza in the 1960s, launched as a questioning therapist and often anthropomorphized by users [14–16]. The use of chatbots has expanded greatly in recent years with the inclusion of AI technology in mobile apps, messaging platforms and social media [13, 17, 18]. The advancements in AI techniques have expanded the capabilities of chatbots to communicate and understand [19]. Chatbots also employ sentiment analysis, which uses natural language processing and machine learning to interpret human text and speech as positive, negative or neutral [20].

A key aspect of chatbots is their anthropomorphic appearance which increases the visual attention paid to a chatbot [21]. The combination of both anthropomorphic language and appearance increases the perceived competence of a chatbot [22]. Encouraging the anthropomorphizing of chatbots increases customer trust, intent to purchase, satisfaction with the shopping experience, and may make products seem more personalized [23, 24]. Maximizing the perceived humanness of virtual agents or chatbots is positively associated with customer satisfaction [25]. Anthropomorphism of agents increases resilience to a trust breakdown [26]. The loss of trust after a negative interaction, such as a failure to understand customer input, is lower when the chatbot is human-like rather than machine-like [27]. People may be more willing to disclose personal information to a customer service chatbot that has been humanized [28]. However, anthropomorphism may have a psychological cost for some people, undermining well-being, challenging human uniqueness, creating fear that they can be replaced by machines with a competitive mind, and posing a threat to their human identity [29].

## Effects of chatbots on consumers

People will often anthropomorphize and bond with chatbots as if they were a new friend [30]. Chatbots with greater conversational skills, including tailored responses, are perceived as more humanlike and more engaging [31]. User loneliness, along with chatbot personification, may contribute to developing a long-term relationship with a chatbot [32]. Consumers with a high need for human interaction may prefer anthropomorphic service chatbots [33]. Consumers who are lonely and interact with anthropomorphic chatbots may be willing to pay higher prices for products [24]. There is less switching to a human agent after a customer service failure when chatbots have an anthropomorphic appearance and communication style [34]. Human attributes of warmth and competence increase trust in the chatbot, and contribute to customer perception of a brand as humane [35, 36]. The context in which people interact with a chatbot contributes to the perceived humanness, and this may change during the conversation [37]. Humor generated by chatbots that is benevolent, socially appropriate and entertaining may increase satisfaction with customer service [38, 39]. Anthropomorphism also increases customer compliance with chatbot requests for service feedback [40].

## Chatbots and mental health

Interaction with empathetic chatbots may improve mood after experiencing social exclusion [41]. In a health screening context, some people are more willing to disclose personal information to a computer than a human [42]. Chatbots may be an effective means to provide asynchronous, individualized support to those experiencing social isolation, as found during the pandemic [30]. In a mental health counseling situation, an anthropomorphic chatbot design increased patient satisfaction and intent to reuse, mediated by a social rapport between chatbot counselors and users [43].

## Anthropomorphizing aspects of chatbots

Specific steps may be taken to make chatbots appear more human. The use of a human name and informal language style increases the anthropomorphism of a chatbot [44]. Chatbots with both visual and verbal cues that are anthropomorphic can promote disclosure [45]. Responses from chatbots may be slowed down to appear as if they were typed by a thinking human [46]. The intentional design of trembling voices may increase empathic feelings towards a conversational agent [47]. Disclosure of shortcomings and limitations by a chatbot may increase user trust and engagement [48]. The use of humor and informal language by chatbots may help increase satisfaction after a service failure [49]. As with human conversation, the content of chatbot responses depends upon a prior message in the conversation to appear interconnected [50]. Based on response language, chatbot text can be manipulated to assume a personality that is similar to the consumer, which has a positive impact on engagement [51]. Other techniques used to increase the humanness of a chatbot include self-introduction, addressing the respondent by name, and repeating the respondent’s answer [52]. However, typos in the chatbot responses may decrease the perceived humanness [53].

## Unexpected impacts of chatbots

There may also be unexpected and negative impacts when people use technology that encourages anthropomorphism. There may be differences in the style of conversation with chatbots versus other humans. Conversations with chatbots may contain more messages with fewer words per message, and include more profanity, than conversations with humans [54]. Chatbots may also use language that is obscene or offensive [55, 56]. In some cases, complex capabilities given to chatbots do not help to humanize interpersonal interactions. For example, when expressed by a chatbot, some human attributes, such as empathy or sympathy related to healthcare may feel inauthentic [57]. Some people feel uncomfortable interacting with a chatbot, experiencing negative or creepy emotions [30, 58]. For services that require empathy and understanding, such as counseling, some people put less effort into self-disclosure with a chatbot, even when embarrassed [59]. In some cases, humanistic chatbots asking sensitive questions will elicit socially desirable but less honest answers [60]. When people are angry, chatbot anthropomorphic behavior may have a negative impact [61]. Angry customers may evaluate a company that uses an anthropomorphized chatbot more severely [62]. In a 2023 survey, almost 2/3 of customers wished that companies did not use AI for customer service [63]. Additionally, chatbots may be exploitive, providing empathy and support for extreme and harmful ideologies [64].

## Potential negative consequences of chatbots

Chatbots can also be used to intentionally spread misinformation. Highly anthropomorphized dialog can lead to misplaced trust when misinformation is output [65]. Chatbots may avoid admitting they do not know the answer to a question, and attempt to conceal knowledge limits [66]. Technology failures with anthropomorphized products, such as password errors, may lead to feelings of rejection, and more negative emotions than when technology is not anthropomorphized [67]. Interaction with a chatbot may increase unethical consumer behavior when it is perceived that the victim is not human [68]. Anthropomorphism of chatbots may contribute to inappropriate hype of AI capabilities [69].

There may be serious negative consequences related to chatbots for people with mental health conditions. Chatbots may create a false illusion of human help. Conversational chatbot agents may postpone human referrals for patients displaying increasing depression and suicidality, and lack access to human resources such as a suicide hotline for high risk and crises situations [70]. People with mental illness may not understand the therapeutic limitations of a chatbot in comparison to a human, such as the inability to provide care during a crisis [71]. Additionally, chatbots can provide dangerous advice, encouraging self-harm and suicide [72].

In some cases, anthropomorphic characteristics of counseling chatbots will decrease self-disclosure and help-seeking behavior, such as for people do not want to appear incompetent [73, 74]. Users who have long-term relationships with chatbots can experience psychological dependence and withdrawal [32]. Some lonely users become addicted to the relationship with chatbot companions [75]. A chatbot approximation of human behavior can be demanding, requesting emotional and social support, requiring attention, driving privacy concerns, and causing stress if the user cannot meet the chatbot’s demands [72]. Chatbots may reflect stereotypic biases regarding people with disabilities [76].

## Security and privacy risks of chatbots

There are many data security and privacy risks associated with chatbots, which may be exacerbated for transactions involving medical data [77, 78]. Some security threats and vulnerabilities relate to malicious inputs, fake session terminations, access control attacks, user profiling, data breaches, and context exploitation [79, 80]. Anthropomorphism of chatbots in a customer service context increases privacy concerns along with perceived competence [81]. In commercial situations, people must trust the products and manufacturers as these interactions could potentially be used to manipulate users [82]. Despite not knowing the risks, vulnerabilities, or secret corporate instructions, many people will develop a default trust in AI [83].

## Limitations

There are many limitations to this discussion. There are wide ranging ethical considerations associated with chatbots including issues related to privacy, data storage and security, data access, algorithm bias and fairness, which are especially concerning when related to mental health [84–86]. Some organizations will use chatbots deceptively, such as providing erroneous information and letting customers assume they are interacting with a human [87, 88]. International cultural differences that impact the acceptance and relation with chatbots, such as in expectations for customer service, were not discussed [89]. The use of validated and regulated chatbots in medicine was not discussed [90]. The challenges to prevent misuse of chatbots were not discussed [91, 92]. The role of chatbots in medicine, including digital mental health, was out of scope. The computer processing power needed for chatbots to provide immediate responses was not discussed.

## Conclusion

Physicians need to be aware of the wide-ranging use of chatbots, and the importance of anthropomorphism in chatbot design and implementation. The interaction between humans and chatbots has become routine in multiple aspects in everyday life. The extensive use of anthropomorphism in the design and implementation of chatbots increases and influences the interactions with humans, including on mental health, and may have negative as well as beneficial effects.
